# Non-Surgical Approaches to the Management of Lumbar Disc Herniation Associated with Radiculopathy: A Narrative Review

**DOI:** 10.3390/jcm13040974

**Published:** 2024-02-08

**Authors:** Ahmed M. El Melhat, Ahmed S. A. Youssef, Moustafa R. Zebdawi, Maya A. Hafez, Lamia H. Khalil, Deed E. Harrison

**Affiliations:** 1 Department of Physical Therapy for Musculoskeletal Disorders and Their Surgeries, Faculty of Physical Therapy, Cairo University, Cairo 12613, Egypt; ahmed.elmelhat@cu.edu.eg; 2 Department of Physical Therapy, Faculty of Health Sciences, Beirut Arab University, Beirut P.O. Box 11-5020, Lebanonzebdawimoustafa@gmail.com (M.R.Z.); mayahafez58@gmail.com (M.A.H.); lamia.khalil02@gmail.com (L.H.K.); 3 Basic Science Department, Faculty of Physical Therapy, Beni-Suef University, Beni-Suef 62521, Egypt; dr.ahmedsameer@pt.bsu.edu.eg; 4 CBP Nonprofit, Eagle, ID 83616, USA

**Keywords:** lumbar disc herniation, discogenic pain, lumbar radiculopathy, conservative management, spinal decompression, extension traction

## Abstract

Lumbar disc herniation associated with radiculopathy (LDHR) is among the most frequent causes of spine-related disorders. This condition is triggered by irritation of the nerve root caused by a herniated disc. Many non-surgical and surgical approaches are available for managing this prevalent disorder. Non-surgical treatment approaches are considered the preferred initial management methods as they are proven to be efficient in reducing both pain and disability in the absence of any red flags. The methodology employed in this review involves an extensive exploration of recent clinical research, focusing on various non-surgical approaches for LDHR. By exploring the effectiveness and patient-related outcomes of various conservative approaches, including physical therapy modalities and alternative therapies, therapists gain valuable insights that can inform clinical decision-making, ultimately contributing to enhanced patient care and improved outcomes in the treatment of LDHR. The objective of this article is to introduce advanced and new treatment techniques, supplementing existing knowledge on various conservative treatments. It provides a comprehensive overview of the current therapeutic landscape, thereby suggesting pathways for future research to fill the gaps in knowledge. Specific to our detailed review, we identified the following interventions to yield moderate evidence (Level B) of effectiveness for the conservative treatment of LDHR: patient education and self-management, McKenzie method, mobilization and manipulation, exercise therapy, traction (short-term outcomes), neural mobilization, and epidural injections. Two interventions were identified to have weak evidence of effectiveness (Level C): traction for long-term outcomes and dry needling. Three interventions were identified to have conflicting or no evidence (Level D) of effectiveness: electro-diagnostic-based management, laser and ultrasound, and electrotherapy.

## 1. Introduction

Low-back pain (LBP) is a broad category of musculoskeletal disorders regarded as one of the primary causes of disability in the general population, with a lifetime incidence of 65–85% of individuals worldwide [1,2]. One of the specific causes of LBP is lumbar intervertebral disc disorders with nerve root irritation. Most individuals affected fall within the age range of 30 to 50 years, with a higher prevalence in men than in women [3].

The intervertebral disc is formed by an inner nucleus pulposus (NP), an outer annulus fibrosus (AF), and the cartilaginous endplates that attach the disc to its vertebrae [4]. The protrusion of disc material into the spinal canal from outside the annular lining is known as herniation. When compared to other regions, the lumbar region is where disc herniation is most commonly observed, especially at the L4-L5 and L5-S1 levels [5]. Lumbar disc herniation associated with radiculopathy (LDHR) is the outcome of the extruded disc material pressing into or contacting the lumbar nerve roots.

A combination of inflammation brought on by local pressure and neurochemical inflammatory substances found in the disc material causes the pain linked to lumbar radiculopathy [5]. Radiculopathy is characterized by numbness, tingling, weakness, and radiating neuropathic type pain, which usually manifests unilaterally. Physical examination findings typically involve decreases or losses in deep tendon reflex in relation to a specific root level, sensory reduction or loss in a specific dermatomal distribution, and muscular weakness in a specific myotomal distribution [6]. Increased symptom severity, a worse prognosis, greater disability, lost productivity, and higher medical expenses have all been linked to nerve root involvement [7].

The treatment of LDHR consists of both non-surgical and surgical procedures. Attempts have been made over the past decade to reduce the necessity for spinal surgery [8]. Most patients prefer conservative treatment over surgery because it carries a lower risk of complications and lower costs [9]. Surgery is recommended when LDHR is severe, lasts longer than six weeks, or fails to improve with conservative treatment [10,11]. Research findings suggest that in the absence of deteriorating neurological symptoms, such as saddle anesthesia, bowel or bladder incontinence, sudden paresis in an extremity, or cauda equina syndrome, non-surgical approaches for lumbar radiculopathy should be considered before surgical approaches [12].

This article aims to review the effectiveness of different non-surgical treatment approaches for the treatment of lumbar disc herniation associated with radiculopathy, providing an insight on the advanced and new approaches in addition to the existing knowledge. An evidence-based treatment approach holds great clinical significance and a high priority in the management of LDHR [13]. Prior to considering surgery, conservative treatments such as patient education, the McKenzie method, mobilization, manipulation, exercise therapy and traction are recommended for patients with LDHR. Other interventions such as neural mobilization, ultrasound, laser, electrotherapy, dry needling and epidural injections are also employed. The effectiveness of each of these non-surgical (conservative care) treatment methods will be reviewed with an emphasis on the contemporary literature in an effort to present an update to the understanding and management of patients suffering from LDHR, and evidence-based ratings and gradings for each of these interventions will be provided at the end for clear and concise clinical management options.

## 2. Pathophysiology of Disc Herniation

The pathophysiology of lumbar disc herniation is crucial to understand in order to develop effective management and treatment strategies, which includes conservative approaches such as physical therapy, and in some cases, surgical intervention. Lumbar disc herniation is a consequence of degenerative changes in the AF, as age-related changes occur, leading to several alterations in the intervertebral disc [14]. These changes include: (1) a reduction in water concentration in the nucleus pulposus, (2) an elevation in type 1 collagen ratio in the nucleus pulposus and inner AF, (3) damage in collagen and extracellular material [15], and (4) an upregulation of matrix metalloproteinase expression (MMP), apoptosis, and inflammatory pathways, leading to the increased breakdown of tissue components, a higher rate of programmed cell death, and an intensified inflammatory response [16]. Eventually, this results in an increased local inflammatory response and mechanical compression affecting the intervertebral disc and compressing on the exiting nerve root.

The intervertebral disc is subject to complex biochemical processes that have a major effect on its mechanical behavior to maintain its integrity. These processes involve an extracellular matrix (ECM) that contains collagen type II, which forms the nucleus pulposus, contributing to its gel-like structure, and proteoglycan, which attracts water molecules to maintain disc hydration. In addition, increases in osmotic pressure help in resisting compression forces and distribute mechanical forces. Simultaneously, collagen fibers give the AF structural integrity, which affects the tensile strength. However, excessive or prolonged mechanical stress can lead to disc degeneration by causing imbalances in extracellular matrix synthesis and degradation. Disc degeneration is facilitated by biochemical interactions, such as the enzymatic breakdown of proteoglycans by matrix MMPs. Elevated MMP activity, for example, may disrupt the balance between matrix synthesis and degradation, which could result in changed mechanical properties and decreased water retention. Recognizing these biochemical complexities highlights the translation of molecular events into mechanical changes, providing significant perspectives for therapeutic approaches aimed at targeting disc health [17,18].

Generally speaking, the progression of degeneration in the annulus fibrosus contributes to the risk of disc herniation [19]. Several mechanisms for disc herniation have been proposed, and these include: (1) nucleus pulposus protrusion through pre-existing AF tears or fissures, (2) AF protrusion due to AF buckling, and (3) mixed herniation types with both NP and AF protrusions [19]. Due to the tissue type and micro-architecture of the AF, the posterior, or the posterolateral region, of the AF contains thinner and incomplete lamellae compared to the anterior AF. These architectural design differences in the posterior and posterior–lateral AF region are the likely explanations for higher failure rates and disc herniation in these regions [19].

Early diagnosis and targeted interventions play a crucial role in significantly improving outcomes and alleviating the impact of lumbar disc herniation on an individual’s quality of life. A herniated disc leads to pressure on the longitudinal ligament and local inflammation, resulting in low back pain caused by irritation. When a disc material exerts and causes a direct contact with the thecal sac or lumbar nerve root, it results in lumbar radicular pain with inflammation and nerve root ischemia. The posterolateral aspect is unsupported by the posterior longitudinal ligament; additionally, on the posterolateral aspect, the annulus fibrosus exhibits a thinner structure, and the proximity of the nerve root makes it more susceptible to herniation of the disc resulting in nerve root compression [4,15].

In lumbar disc herniation (LDH), the constriction of the space surrounding the thecal sac is caused by multiple factors. Among these are the protrusion of the intervertebral disc through an undamaged annulus fibrosus, preserving the continuity of the disc space; there is also extrusion of the nucleus pulposus through the annulus fibrosus, and the obliteration of disc space continuity with the isolation of a free fragment [4]. Each of these mechanisms contributes to a reduction in available space around the thecal sac, potentially leading to nerve compression and associated symptoms.

## 3. Classification of Lumbar Disc Herniation

The classification of lumbar disc herniation is based on several factors such as location, extent of nerve root involvement, clinical presentation, severity and direction. Lumbar disc herniation is classified into disc bulge, protrusion, extrusion and sequestration. Disc bulge occurs when the circumference of the disc exceeds beyond the regular margins of the vertebral body while maintaining the circumferences of the disc, causing asymmetric bulging mainly on one side. Disc protrusion is recognized when the base width of the protrusion is wider than the diameter of the disc material that is herniated, and it projects beyond the normal disc margins without damage to the annulus fibrous. Disc extrusion occurs when there is damage of the annulus fibrous, allowing the nucleus pulposus to extend beyond the normal margins [4]. In situations where the annulus structure undergoes complete disruption, there exists the potential for the nucleus content to extrude outside the disc space, resulting in the migration of a nucleus pulposus fragment called sequestration [14].

Certain patients with lumbar disc extrusion or protrusion may not experience symptoms, a condition known as “asymptomatic disc herniation”, as the presence of disc abnormalities on imaging does not correlate with the presence or severity of the symptoms [20]. Many factors contribute to the expression of symptoms, including different pain thresholds, as individual variations in pain perception can influence whether a herniated disc becomes symptomatic. Moreover, inflammatory processes contribute to symptoms; in addition, the body’s immune response to disc material that leaks out in herniation leads to irritation and swelling [21]. Furthermore, not all disc tissue will cause radicular pain that radiates along nerve pathways, as some individuals may experience localized back pain without radiation, and will experience discogenic pain rather than nerve compression [22]. Furthermore, in some cases, the body’s natural healing process can lead to a resorption or reduction in size of a herniated disc over time; this can occur without the individual ever experiencing significant symptoms [23]. All of the aforementioned variables contribute to the variety in the expression of symptoms among individuals with similar disc herniations. A comprehensive classification system contributes to improving patient outcomes and ensuring that therapeutic strategies align with the unique characteristics of lumbar disc herniation in each individual case.

**Figure 1:**
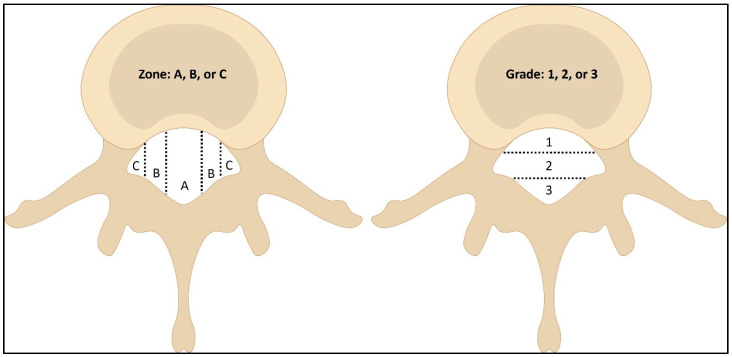
Lumbar herniated nucleus pulposus HNP classification using the Michigan State University (MSU) system for a combination of size and location of disc herniation. The MSU uses magnitude grades 1 to 3 and medial to lateral herniation location from zone A to zone C. Adapted from Mysliwiec LW et al. [24].

Many factors, including pain thresholds, inflammation, and the body’s ability to adapt, contribute to the variety in the symptoms among individuals with similar disc issues. A comprehensive classification system contributes to improving patient outcomes and ensuring that therapeutic strategies align with the unique characteristics of lumbar disc herniation in each individual case. The Michigan State University (MSU) classification for lumbar disc herniation is recommended herein as it is a simple and reliable method to objectively measure herniated lumbar discs [24]. The MSU provides classifications of disc herniation magnitude as 1, 2, or 3, and provides a herniation location of A, B, or C; the MSU has excellent reliability for its classifications [24]. Figure 1 depicts the MSU classification system.

## 4. Conservative Treatment Approaches

### 4.1. Patient Education and Self-Management

Patient education is considered one of the key elements emphasized by physical therapists, which include recommendations such as avoiding complete bed rest and avoiding strenuous activities. This fundamental guidance plays a crucial role in promoting recovery and well-being, especially in the presence of worsening and persistent pain that warrants consultation with a physical therapy. When a patient reaches cauda equina symptoms, they should seek medical help urgently, and the treatment time will depend on prognosis and outcomes from the initial symptoms [14,15]. Patient education should be tailored to individual needs, and involves collaboration between physical therapist and the patients themselves. Providing comprehensive information empowers individuals to take an active role in maintaining spine health and preventing the deterioration of the symptoms.

A conservative treatment plan should include educating the patients about life style modifications and home exercise programs, along with ergonomics instruction with active physical therapy [25]. These are in addition to maintaining healthy weight, as excess weight can contribute to increased pressure on lumbar disc, and encouraging a balanced exercise routine that includes activities to strengthen the core muscles, as to provide better support for the spine. Furthermore, proper approaches to lifting objects should be conveyed, with proper body posture that minimizes the strain on the lumbar spine intervertebral disc [26]. Self-management and patient education should also be included, with other physical activities such as exercises and manual therapy.

### 4.2. Electrodiagnosis-Based Management

In radiculopathy, the direction of the nerve root compression is sensitive. Direction-sensitive exercise (DSE) therapy can help decompress the nerve root. Testing the H-reflexes in the “static” and “dynamic” protocols can help determine which way the nerve is compressing or decompressing. The compression posture is referred to as the undesired spinal posture (USP), while the decompression posture is known as the optimum spinal posture (OSP) [27]. The soleus H-reflex, which is regarded as the best electrophysiological test for nerve root function, has been used to establish a method for determining the proper spine posture during manipulation [28]. Because of the preserved OSP, this method reduces impaired disc herniation and appears to lessen neural impingement as well as neural irritation and inflammation. The gradual alleviation of symptoms is most likely caused by the axons of the motor and sensory nerves being decompressed [27]. It is demonstrated that this approach is effective for treating radiculopathy.

### 4.3. Mechanical Diagnosis and Therapy

The Mechanical Diagnosis and Therapy (MDT) approach, developed by McKenzie for treating low back and associated leg pain, aims to restore compromised spinal segments to normal function and alleviate pain [29]. Changes in the location of low-back and radiating pain following repeated spinal motions were first described by McKenzie. This method involves employing repeated lumbar movements in specific directions, such as flexion or extension, to induce a positive change in the patient’s condition manifested as the centralization phenomenon, where pain migrates from a distal (peripheral) to a proximal (central) location on the spinal midline [30]. Utilizing McKenzie exercises that involve repeated lumbar motions in the direction that produces centralization is associated with better results, leading to immediate symptom improvement by effectively eliminating and preventing the recurrence of the patients’ pain [31,32,33].

### 4.4. Mobilization and Manipulation Techniques

Mobilization and manipulation involve a spectrum of adeptly administrated motions performed at different speeds and amplitudes, either within the range of, or at the end range of, the motion of the joint. Thrust intervention involves low-amplitude and high-velocity maneuvers. In the context of lumbar disc radiculopathy, both thrusting and non-thrusting techniques are recognized for their non-invasive nature, making them viable approaches for application. Additionally, spinal manipulation techniques are considered safe and effective in treating lumbar disc herniation.

Manual therapy, including mobilization and manipulation, can enhance neuromuscular coordination by addressing joint restrictions and promoting optimal movement patterns. Manual therapy contributes to improving coordination in the lumbar region [34]. Moreover, mobilization and manipulation offer short-term relief from pain associated with lumbar disc problems, contributing to enhancements of function and quality of life. By enhancing joint flexibility and reducing stiffness and discomfort, mobilization makes daily activities more manageable.

Mulligan’s method is hypothesized to alleviate nerve compression by enhancing vertebral rotation and creating increased space between intervertebral discs. As this study has mentioned, Mulligan’s technique for lumbar radiculopathy has the ability to alleviate nerve compression with increased vertebral rotation within intervertebral space. Moreover, the management of lumbar disc herniation, combined with spinal mobilization with leg movement, along with the progressive inhibition of neuromuscular structures, has been found to be effective in radiculopathy [35,36]. Mulligan’s techniques have shown effectiveness in reducing pain, enhancing range of motion, and producing positive functional outcomes for specific musculoskeletal conditions.

Recently, Lizis and colleagues [37] presented a pilot randomized trial comparing the short-term effectiveness of Kaltenborn–Evjenth Orthopedic Manual Therapy (KEOMT) vs. Kinesiotherapy (KIN) on the quality of life and pain in patients with LDHR. Eighty participants between the ages of 40 and 70 years old were included, and both groups completed 10 treatments sessions over the course of 5 weeks; outcome measures included quality of life and pain scores. At the end of the 10 treatment sessions, statistically significant differences were identified favoring the KEOMT group; they concluded that patients receiving KEOMT achieved better improvements in patients suffering from chronic LDHR [37].

### 4.5. Exercise Therapy

Exercise training enhances muscle power, strength and endurance, especially when targeting deep muscles of the trunk such as transverse abdominals and multifidus, as it helps improve the coordination and stability of the trunk region. Engaging in dynamic physical activities and integrating stretching regimens has been observed to elicit analgesic effects in the context of disc herniation, highlighting the significance of avoiding prolonged periods of complete rest. According to Huber et al. (2011) [38], a group received muscle strengthening and endurance exercises was compared to a group receiving only reductions in activity and loading, and the first group showed significant improvements and reductions in symptoms.

Regardless of the mechanism of injury in herniated discs, weak core muscles and reduced spinal stability result in a significant delay in healing. Core-strengthening exercises are required in the rehabilitation of lumbar disc herniation to provide an entire range of movements, as this area acts as the functional center of the kinetic chain between upper and lower extremities [39]. Enhancing core muscle strength is an essential factor to increase intra-abdominal pressure during spinal movements, which will contribute to stability and reduce the load on the lumbar spine.

Lumbar stabilization exercises and manipulation are effective in reducing symptoms of lumbar disc herniation by strengthening the muscles that support the lumbar region and improving the stability. Stabilization exercises provide controlled balance in pelvic movement, and enhance the stability and mobility of the sacroiliac joint. Consequently, they increase pelvic and back movement, which has a positive effect on lumbar discs [40]. This approach aligns with the findings of Ye et al. (2015) [41], through which they observed that the activation of trunk muscles through specific exercises plays a crucial role in improving back pain and reducing instability in patients with lumbar disc herniation.

### 4.6. Traction-Distraction and Flexion

Traction is the most common modality used in non-surgical spinal decompression (NSD) therapy. The NSD therapy is intended to offer a motorized segmental distraction for a predetermined amount of time, thus leading to physical changes in the disc [42,43]. Traction plays a crucial role in addressing lumbar disc herniation by increasing the space between vertebrae and stretching the posterior longitudinal ligament. This action generates a pulling force that directs the herniated disc toward the center of the joint [44]. Simultaneously, traction contributes to an enhancement in intervertebral space and the opening of foramina, thereby improving disc height. Furthermore, Ljunggren et al. [45] investigated 49 patients with chronic LDHR by randomly assigning them to either an NSD group or a manual traction group, where intervention was applied by the same therapist for fidelity. Interventions were applied over the course of 1 week using a blinded assessment at three time intervals: immediately after the traction period, after two weeks of follow-up, and at three months. A two year follow-up was performed with no recurrence of symptoms reported. While both types of traction methodology were found equally effective, manual traction (using the Kaltenborn–Evejnth method) was recommended due to its simplicity [45].

It is interesting that recent international guidelines [46] and consensus initiatives [47] continue to dismiss the evidence that promotes the use of lumbar spine traction for the management of low-back pain with or without radiculopathy due to disc herniation; in fact, spinal traction is rather emphatically listed as a procedure without supporting evidence and not to be employed [46,47]. Interestingly, these consensus documents/opinions [46,47] are in opposition to four recent systematic reviews with meta-analyses [48,49,50,51], and are supported by more recent randomized trials [52], which establish the clinical utility and early effectiveness of traction for LDHR [48,49,50,51]. For example, these meta-analyses have all concluded that NSD traction is effective for relieving back and leg pain intensity [48,49,50,51] and disability [50] due to lumbar disc herniation. However, Cheng et al. [48] concluded that the effects of NSD were only significant in terms of short-term outcomes. While Wang and colleagues identified that NSD traction may not notably affect the range of motion in the lumbar spine, they found that it does prove effective in relieving low-back and leg pain, as well as overall disability, in patients with lumbar disc herniation [50].

Thus, the multifaceted effects of traction underscore its therapeutic potential in addressing lumbar disc herniation, encompassing structural improvements and neural benefits. Both supine and prone lying positions are utilized in the application of mechanical traction. While therapists typically prefer the supine position [53], it is noteworthy that the prone lying position demonstrates a notable decrease in muscle tension and mild muscular activation compared to the supine position [54], which may facilitate more pronounced intervertebral separation. Consequently, the prone lying position could be regarded as more favorable compared to the supine lying position when addressing chronic lumbosacral radiculopathy [55,56,57].

#### Extension Traction or Lordosis Enhancing

One of the most common radiographic findings in populations with chronic lower-back pain is a concomitant reduction in the normal lumbar lordotic curvature, which is consistent in those with disc injuries as well [58]. Intuitively, then, low-back disorder patients who concurrently have lumbar hypo-lordosis would require a type of traction that enhances the natural lumbar lordosis or lumbar extension traction (LET). Problematically, the classically applied NSD traction is known to flatten/straighten out the lumbar lordosis, and while effective in many cases, this may not be the optimum for those with significant hypo-lordosis of the lumbar spine [59,60]. While spinal traction has been around for literally hundreds of years, it was not until 2002 that the first clinical trial on LET was published; though this was done using patients with chronic low-back pain (CLBP) without radiculopathy [61]. This 2002 clinical trial demonstrated, in CLBP patients with hypolordosis, that routine improvements in the lumbar curvature are achievable [61].

Since the original trial outlining the effectiveness of the LET approach for lumbar hypolordosis, three more randomized controlled trials have documented that superior outcomes occur in mechanical LBP and sciatic patients receiving LET as part of comprehensive physiotherapeutic programs versus those who receive the physiotherapy without the extension traction [61]. The randomized trial by Moustafa et al. [62] demonstrated that patients with CLBP and radiculopathy due to disc herniation that were treated with LET had better long-term outcomes in back pain, disability, and lumbar flexion and extension kinematics. Furthermore, the LET group showed improved lumbar lordosis and a significant improvement in the H-reflex, likely due to reducing stress on neural tissue from the increased lumbar lordosis [62]. Similarly, in the most recent randomized trial using a modified form of LET adapted to a spinal distraction table, Lee and colleagues [63] identified (after 15 traction sessions applied over the course of 5 weeks) that the LET group experienced better short-term improvements in pain, function and disc morphology in 20 patients with discogenic radiculopathy.

The two randomized trials on LET for discogenic radiculopathy with CLBP offer good preliminary evidence for its clinical utility, though only the Moustafa et al. trial had a 6-month follow-up [62,63]. However, two case reports have also been published documenting the clinical efficacy of LET methods applied to patients with CLBP and discogenic radiculopathy and loss of the lumbar lordosis [64,65]. Thus, while the strength of the evidence on LET is preliminary, there is enough evidence to recommend this as a viable treatment solution for specific disc herniation patients with loss of the lumbar lordosis without other complications such as spinal stenosis [59,60,61,62,63,64,65,66]. It is noteworthy that LET procedures and investigations are not discussed in the consensus guidelines mentioned above [46,47].

### 4.7. Neural Mobilization

The implementation of a neural mobilization with a motor control exercise program has been shown to reduce neural mechanosensitivity and neuropathic symptoms. However, the neural mobilization technique is efficient for managing pain and disability only via short-term treatment [67,68]. As a neural mobilization added to a physical therapy exercise program, or combined with non-thrust mobilization with electrotherapy, it can improve the outcomes and result in a reduction in pain and disability.

### 4.8. Laser and Ultrasound

The mode of action of a laser is through tissue stimulation, producing analgesic and anti-inflammatory effects. Low-level laser therapy, administered at a wavelength of 830 nm and a dose of 3 J/point for discogenic lumbar radiculopathy, demonstrated effectiveness in significantly improving trunk movements, and reducing pain intensity and associated functional disability [69]. Ultrasound (US) produces various effects through its thermal impact, including enhanced nerve transmission speed, the elongation of collagen tissue, increased blood flow rate, decreased pain threshold, and the alleviation of muscle spasms [70]. A high-quality trial provided moderate evidence that there are no significant differences between laser and mechanical traction, between ultrasound and mechanical traction, and between laser and ultrasound for back pain intensity, leg pain intensity, or function at short- and intermediate-term follow-ups [71].

### 4.9. Electrotherapy

Electrotherapy involves the application of electrical energy in medical treatment. In order to increase the range of motion and decrease the degree of radicular pain associated with LDHR, using either transcutaneous electrical nerve stimulation (TENS), interferential (IF) stimulation, or a combination of pulsed ultrasound and IF current (CTPI) is sufficient. Because of the induced current’s higher penetration potency, CTPI tends to be the most efficient modality out of the three [72]. Electrical stimulation therapy aids in reducing pain intensity and alleviating clinical symptoms and signs in individuals with LDHR, hence promoting patients’ rehabilitation.

### 4.10. Dry Needling

A myofascial trigger point is a hypersensitive area within a tight skeletal muscle, causing pain when compressed. The multifidus muscle, one of the paraspinal muscles, is exposed to prolonged contraction, causing radicular pain due to narrowing in the intervertebral foramina and disc space. Dry needling, in conjunction with standard physical therapy such as thermal modalities, TENS, ultrasound and McKenzie exercises, leads to significant improvements compared to standard physical therapy alone [73].

According to Dernek et al. (2018) [74], patients undergoing dry needling and an exercise program to treat trigger points with lumbar disc radiculopathy showed a decrease in pain and enhanced functional status compared to an exercise program alone. The trigger points are hyperirritable spots in the muscle, and dry needling helps in releasing or deactivating trigger points within the muscle.

Another theory suggests that dry needling leads to temporary damage to muscle fibers by disrupting sensory and motor components in the nerve endings [75]. Moreover, it releases local intracellular potassium, preventing nerve depolarization. Additionally, it releases endorphins, supporting the gate control theory of pain. This technique involves using dry needling to penetrate the skin and stimulate the underlying myofascial trigger point and muscular tissue, in order to manage pain.

### 4.11. Epidural Injection

Epidural injection is one of the procedures that is used to treat the irritation and inflammation of the spinal nerve root caused by intervertebral disk herniation. Moreover, anesthetic and corticoids injections are employed as blockade treatments of the nerve root to reduce the inflammatory response and decrease the volume of disc herniation.

Epidural corticosteroid injections are used to relieve he pain associated with lumbar disc herniation within a short-term period (2 to 4 weeks) [15,76]. There is significant support for their efficacy in reducing the nerve root irritation associated with lumbar disc issues, particularly when compared to epidural injections used to treat the irritation caused by spinal stenosis.

## 5. Discussion

The current comprehensive review highlights the significance of non-surgical interventions for individuals experiencing lumbar disc herniation with associated radiculopathy. In 2023, the World Health Organization (WHO) released consensus guidelines on chronic low-back pain (CLBP) disorders [47]. These guidelines emphasize the importance of non-surgical interventions, aligning with the principles of the International Classification of Functioning, Disability, and Health (ICF) from the Academy of Orthopaedic Physical Therapy of the American Physical Therapy Association, and the American Spine Society also contributed to this comprehensive review, underscoring the significance of non-surgical approaches in managing individuals with lumbar disc herniation and associated radiculopathy. The collaboration between these organizations reinforces the commitment to evidence-based clinical practice guidelines, and highlights the collective effort to address the complex challenges associated with LBP, particularly in the context of lumbar disc herniation and radiculopathy [77,78]. The featured interventions encompass a spectrum of modalities, including patient education, mechanical diagnosis and therapy (MDT), mobilization and manipulation, exercise therapy, traction, neural mobilization, ultrasound and laser, electrotherapy, and epidural injection [47,77,78].

While therapists incorporate standard education approaches, it is crucial to recognize that education alone is not a stand-alone treatment. Recommendations typically include guidance on physical activity and the promotion of an active lifestyle. For patients with acute and chronic LBP with or without radiculopathy, therapists may implement MDT to alleviate pain and reduce disability. Therapists should employ thrust or non-thrust joint mobilization techniques to decrease pain and disability in patients with acute and chronic low-back pain (LBP). This approach is also applicable for patients with chronic LBP with leg pain. Additionally, spinal manipulation stands as an option for symptomatic relief in individuals experiencing LDHR [47,77,78]. In the domain of exercise training interventions, the evidence is inconclusive regarding the use of structured exercise regimens as stand-alone treatments. However, for patients experiencing mild to moderate symptoms of LDH, a course of structured exercise is a viable option. Thus, in the management of chronic LBP, therapists are advised to incorporate active treatments such as stretching and strength training, steering away from reliance on standalone educational programs [47,77,78].

According to the consensus guidelines reviewed, regarding mechanical traction, the available data are insufficient to either endorse or discourage its usage in chronic LDHR treatment, considering its limited effectiveness when combined with other therapies [47,77,78]. However, our review questions the sparse recommendation of traction due to the fact that four systematic reviews with meta-analysis have indicated its short-term effectiveness for the management of patients suffering from LDHR [48,49,50,51]. Furthermore, these guidelines [46,47,77,78] ignore the evolving literature on using LET for the restoration of lumbar lordosis in LDHR [59,60,61,62,63,64,65,66]. Concerning neural mobilization and dry needling, these modalities may be synergistically integrated with other therapies to achieve short-term pain relief and reduce disability. Nevertheless, the available data are inadequate to validate the utilization of ultrasound, low-power lasers, or electrical stimulation. For individuals seeking short-term pain relief, the consideration of an epidural injection is recommended [47,77,78].

This collective insight supports the notion that, in order to achieve the best possible outcomes for patients, a customized and evidence-based approach is necessary to address the various complexities associated with LDHR.

## 6. Evidence-Based Practice

Patients undergoing conservative treatment typically experience benefits in about 80% of cases within four to six weeks [79]. However, in cases of more severe pain, nerve block becomes an option, and surgical procedures are considered when conservative management fails or the neurological symptoms progress. Furthermore, conservative and surgical treatments aim to achieve similar goals after two years, with surgical procedures providing faster relief from pain [15,25]. Other studies support the idea that surgical intervention helps in the faster relief of symptoms and a rapid return to normal function [5]. Conservative and surgical approaches are considered equally attainable for treating radiculopathy, depending on the stage and symptoms reported by the patient. Moreover, for a long-term lumbar radiculopathy symptom, consistently, conservative and surgical management can reduce the severity of the symptoms and improve the quality of life.

According to Kuligowski et al. (2021) [80], a multi-disciplinary approach is more effective in treating lumbar disc radiculopathy, involving traction, spinal mobilization and the activation of core muscles. In Table 1, the level and strength of evidence of interventions for the management of LDHR is presented. The description of each level of evidence detailed in Table 2 and Table 3 describes the overall grades of recommendations and their relative strengths [77]; these are slightly modified in our current review. Specifically, we have identified the following interventions to have moderate proof (Level B) of utility in the conservative treatment of LDHR: patient education and self-management, McKenzie method, mobilization and manipulation, exercise therapy, traction (short-term outcomes), neural mobilization, and epidural injections. Two interventions were identified to have weak evidence of effectiveness (Level C): traction for long-term outcomes and dry needling. Three interventions were identified to have conflicting or no evidence (Level D) of effectiveness: electro-diagnostic-based management, laser and ultrasound, and electrotherapy.

The importance of tailoring conservative treatment plans to individual patient profiles should be the priority for clinicians managing patients with acute or chronic disorders suffering from LDHR. Patient variables, including their specific symptoms (type, nature, intensity disability, etc.), dietary and nutritional habits, and lifestyle modifications are each important in understanding a given individual’s response to treatments. The interventions with moderate evidence (Level B) should be the primary methods used in the first-line management of patients with LDHR. However, when a patient presents with an abnormal radiographically verified loss of the lumbar lordosis coupled with chronic LDHR, extension traction (Level C evidence) should be considered along with NSD as a viable treatment intervention, as this has been shown to improve long-term outcomes in this specific subgroup of patients [62]. In general, clinicians should steer away from interventions rated as Level D (electro-diagnostic-based management, laser and ultrasound, and electrotherapy), and only base these therapies on evidence of subject-specific needs and characteristics if they can be identified.

### Gaps in Knowledge

While providing a comprehensive overview of existing knowledge and introducing advanced treatment techniques, there remains a call for continued investigations to address the existing gaps in the field. More research should investigate methods that focus on the objective measurement of lumbar disk herniation, and develop treatment methods based on MRI findings. By focusing on these gaps, future research has the potential to expand therapeutic strategies, further advancing our ability to optimize patient care and outcomes in the treatment of LDHR.

## 7. Conclusions

This review undertakes a thorough examination of recent clinical research, delving into non-surgical strategies for lumbar disc herniation and radiculopathy (LDHR), which is considered among the most frequent musculoskeletal and neurological complaints to be examined. Therapists armed with the insights from this review can proficiently navigate the complexities of lumbar disc herniation with radiculopathy, tailoring conservative treatment approaches to individual patient needs. By customizing therapy regimens based on unique requirements, encompassing overall health and symptom severity, therapists are expected to offer optimal relief and enhanced functionality, sparing patients from the necessity of surgical intervention. Specifically, we identified the following interventions to show moderate evidence (Level B) of their applicability for the conservative treatment of LDHR: patient education and self-management, the McKenzie method, mobilization and manipulation, exercise therapy, traction (short-term outcomes), neural mobilization, and epidural injections. Two interventions were identified to have weak evidence of effectiveness (Level C): traction for long-term outcomes and dry needling. Three interventions were identified to have conflicting or no evidence (Level D) of effectiveness: electro-diagnostic based management, laser and ultrasound, and electrotherapy. The interventions with Levels B and C evidence should be provided to patients suffering from LDHR based on individual needs and characteristics.

## Figures and Tables

**Table 1:** Visualization of the level and strength of evidence for interventions addressing LDHR.

Intervention	Description	Level of Evidence
**Patient Education and Self-Management**	A comprehensive area of healthcare that focuses on providing information and support to patients, empowering them to make informed decisions about their health and well-being. The patient education domain encompasses various elements to enhance patients’ understanding of their medical conditions, treatment options, and self-care strategies.	Level B (II)
**Electro-Diagnosis-Based Management**	A method for determining the proper spine posture during manipulation that can help decompress the nerve root.	Level D
**McKenzie Method**	A method of classification based on variations in symptoms associated with low-back pain (and/or lower extremity in response to repeated direction-specific movements of the lumbar spine). The findings are employed to categorize patients into different syndromes (derangement, dysfunction, or postural), directing the choice of treatment approach.	Level B (II)
**Mobilization and Manipulation**	Mobilization is a manual therapy technique that involves passive movement applied to a joint or soft tissue to restore or enhance range of motion. Manipulation, also known as high-velocity, low-amplitude thrust (HVLA), is a manual therapy technique involving a quick, controlled force applied to a joint beyond its passive range of motion. Both are used to address musculoskeletal issues, improve joint mobility and reduce pain.	Level B (II)
**Exercise Therapy**	Exercise therapy is a crucial component of the management and rehabilitation of disc herniation. The primary goals of exercise therapy for disc herniation include improving flexibility, strength, posture, and overall function, while also addressing pain and preventing future issues.	Level B (II)
**Traction**	A treatment involving the application of manual or mechanical forces with the aim of stretching and separating the spine; or, in the case of LET, the goal is restoring the natural lumbar lordosis.	Level B (I) Short term; Level C Long term
**Neural Mobilization**	A therapeutic intervention involving systematic and controlled movements of neural tissues, including nerves, to alleviate neural tension, improve nerve glide, and optimize neurophysiological function.	Level B (II)
**Laser and Ultrasound**	Therapeutic modalities used in physiotherapy.	Level D
**Electrotherapy**	Electrotherapy modalities entail introducing physical energy into a biological system, leading to specific physiological changes utilized for therapeutic advantages.	Level D
**Dry Needling**	A technique that utilizes thin, solid needles to penetrate the skin and stimulate underlying myofascial trigger points, providing relief from muscle tension and pain, and promoting muscle function.	Level C (III)
**Epidural Injection**	Epidural injection for nerve block is a common medical procedure used to alleviate pain and inflammation associated with conditions such as disc herniation. This intervention involves the injection of medication into the epidural space, which is the space surrounding the spinal cord and nerve roots.	Level B (II)

**Table 2:** Level of Evidence.

**I**	High-quality diagnostic studies, prospective study, randomized–controlled trial, or systematic review and/or meta-analysis.
**II**	Lesser-quality diagnostic studies, prospective studies, randomized–controlled trial, or systematic review (weak diagnostic criteria, less than 80% follow up, no blinding).
**III**	Case–control studies or retrospective studies.
**IV**	Case series.
**V**	Expert opinion.

**Table 3:** Strength of evidence.

Grades of Recommendation	Strength of Evidence
**A (Strong Evidence)**	The suggestion is backed by a majority of level I and/or level II studies, with a requirement for at least one level I study.
**B (Moderate Evidence)**	The recommendation is substantiated by either a well-executed randomized–controlled trial of high quality or a majority of exclusively level II studies. This encompasses studies with brief follow-up periods (e.g., 3 months or less) and smaller sample sizes (e.g., fewer than 100 participants).
**C (Weak Evidence)**	The recommendation is backed by only one level II study.
**D (Conflicting or No Evidence)**	Level I and/or level II studies either contradict in their conclusions or offer no evidence of benefit.

## Data Availability

Not applicable.

## List of Abbreviations

LBP	Low-back pain
CLBP	Chronic low-back pain
NP	Nucleus pulposus
AF	Annulus fibrosus
LDHR	Lumbar disc herniation associated with radiculopathy
LDH	Lumbar disc herniation
MMP	Matrix metalloproteinase expression
ECM	Extracellular matrix
DSE	Direction-sensitive exercise
USP	Undesired spinal posture
OSP	Optimum spinal posture
MDT	Mechanical diagnosis and therapy
NSD	Non-surgical spinal decompression
LET	Lumbar extension traction
US	Ultrasound
TENS	Transcutaneous electrical nerve stimulation
IF	Interferential
CTPI	Combination of pulsed ultrasound and interferential current

## References

[1] Ozturk B., Gunduz O.H., Ozoran K., Bostanoglu S. (2006). Effect of continuous lumbar traction on the size of herniated disc material in lumbar disc herniation. Rheumatol. Int..

[2] Satpute K., Hall T., Bisen R., Lokhande P. (2019). The Effect of Spinal Mobilization with Leg Movement in Patients with Lumbar Radiculopathy—A Double-Blind Randomized Controlled Trial. Arch. Phys. Med. Rehabil..

[3] Schoenfeld A.J., Laughlin M., Bader J.O., Bono C.M. (2012). Characterization of the Incidence and Risk Factors for the Development of Lumbar Radiculopathy. J. Spinal Disord. Tech..

[4] Waxenbaum J.A., Reddy V., Futterman B. (2023). Anatomy, Back, Intervertebral Discs. StatPearls.

[5] Schoenfeld A.J., Weiner B.K. (2010). Treatment of lumbar disc herniation: Evidence-based practice. Int. J. Gen. Med..

[6] Tamarkin R.G., Isaacson A.C. (2023). Electrodiagnostic Evaluation of Lumbosacral Radiculopathy. StatPearls.

[7] Fritz J.M., Thackeray A., Childs J.D., Brennan G.P. (2010). A randomized clinical trial of the effectiveness of mechanical traction for sub-groups of patients with low back pain: Study methods and rationale. BMC Musculoskelet. Disord..

[8] Eichen P.M., Achilles N., Konig V., Mosges R., Hellmich M., Himpe B., Kirchner R. (2014). Nucleoplasty, a minimally invasive procedure for disc decompression: A systematic review and meta-analysis of published clinical studies. Pain Physician.

[9] Hahne A.J., Ford J.J., McMeeken J.M. (2010). Conservative management of lumbar disc herniation with associated radiculopathy: A systematic review. Spine.

[10] Alentado V.J., Lubelski D., Steinmetz M.P., Benzel E.C., Mroz T.E. (2014). Optimal Duration of Conservative Management Prior to Surgery for Cervical and Lumbar Radiculopathy: A Literature Review. Glob. Spine J..

[11] Kreiner D.S., Hwang S.W., Easa J.E., Resnick D.K., Baisden J.L., Bess S., Cho C.H., DePalma M.J., Dougherty P., Fernand R. (2014). An evidence-based clinical guideline for the diagnosis and treatment of lumbar disc herniation with radiculopathy. Spine J..

[12] Berry J.A., Elia C., Saini H.S., Miulli D.E. (2019). A Review of Lumbar Radiculopathy, Diagnosis, and Treatment. Cureus.

[13] Singh V., Malik M., Kaur J., Kulandaivelan S., Punia S. (2021). A systematic review and meta-analysis on the efficacy of physiotherapy intervention in management of lumbar prolapsed intervertebral disc. Int. J. Health Sci..

[14] De Cicco F.L., Camino Willhuber G.O. (2023). Nucleus Pulposus Herniation. StatPearls.

[15] Al Qaraghli M.I., De Jesus O. (2023). Lumbar Disc Herniation. StatPearls.

[16] Martin M.D., Boxell C.M., Malone D.G. (2002). Pathophysiology of lumbar disc degeneration: A review of the literature. Neurosurg. Focus.

[17] Martins D.E., de Medeiros V.P., Wajchenberg M., Paredes-Gamero E.J., Lima M., Reginato R.D., Nader H.B., Puertas E.B., Faloppa F. (2018). Changes in human intervertebral disc biochemical composition and bony end plates between middle and old age. PLoS ONE.

[18] Żak M., Pezowicz C. (2021). Effect of overload on changes in mechanical and structural properties of the annulus fibrosus of the intervertebral disc. Biomech. Model. Mechanobiol..

[19] Zhou M., Theologis A.A., O’connell G.D. (2023). Understanding the etiopathogenesis of lumbar intervertebral disc herniation: From clinical evidence to basic scientific research. JOR Spine.

[20] Chadha M., Srivastava A., Kumar V., Tandon A. (2022). Disc Degeneration in Lumbar Spine of Asymptomatic Young Adults: A Descriptive Cross-Sectional Study. Indian J. Orthop..

[21] Ye F., Lyu F., Wang H., Zheng Z. (2022). The involvement of immune system in intervertebral disc herniation and degeneration. JOR Spine.

[22] Fujii K., Yamazaki M., Kang J.D., Risbud M.V., Cho S.K., A Qureshi S., Hecht A.C., Iatridis J.C. (2019). Discogenic Back Pain: Literature Review of Definition, Diagnosis, and Treatment. JBMR Plus.

[23] Hornung A.L., Baker J.D., Mallow G.M., Sayari A.J., Albert H.B., Tkachev A., An H.S., Samartzis D. (2023). Resorption of Lumbar Disk Herniation. JBJS Rev..

[24] Mysliwiec L.W., Cholewicki J., Winkelpleck M.D., Eis G.P. (2010). MSU Classification for herniated lumbar discs on MRI: Toward developing objective criteria for surgical selection. Eur. Spine J..

[25] Gugliotta M., da Costa B.R., Dabis E., Theiler R., Jüni P., Reichenbach S., Landolt H., Hasler P. (2016). Surgical versus conservative treatment for lumbar disc herniation: A prospective cohort study. BMJ Open.

[26] Akca N.K., Aydin G., Gumus K. (2017). Effect of body mechanics brief education in the clinical setting on pain patients with lumbar disc hernia: A randomized controlled trial. Int. J. Caring Sci..

[27] Sabbahi M.A., Ovak-Bittar F. (2018). Electrodiagnosis-based management of patients with radiculopathy: The concept and application involving a patient with a large lumbosacral disc herniation. Clin. Neurophysiol. Pract..

[28] Sabbahi M., Ovak-Bittar F., Abdilahi A. (2015). Low back pain: Manipulate and mobilize in the right direction based on EMG studies. Physiotherapy.

[29] McKenzie R.A. (2003). The Lumbar Spine: Mechanical Diagnosis and Therapy.

[30] Rabin A., Shmushkevich Y., Kalichman L. (2019). Initial pain and disability characteristics can assist the prediction of the centralization phenomenon on initial assessment of patients with low back pain. J. Man. Manip. Ther..

[31] Browder D.A., Childs J.D., Cleland J.A., Fritz J.M. (2007). Effectiveness of an Extension-Oriented Treatment Approach in a Subgroup of Subjects with Low Back Pain: A Randomized Clinical Trial. Phys. Ther..

[32] Halliday M.H., Pappas E., Hancock M.J., Clare H.A., Pinto R.Z., Robertson G., Ferreira P.H. (2016). A Randomized Controlled Trial Comparing the McKenzie Method to Motor Control Exercises in People with Chronic Low Back Pain and a Directional Preference. J. Orthop. Sports Phys. Ther..

[33] Petersen T., Larsen K., Nordsteen J., Olsen S., Fournier G., Jacobsen S. (2011). The McKenzie method compared with manipulation when used adjunctive to information and advice in low back pain patients presenting with centralization or peripheralization: A randomized controlled trial. LWW.

[34] Zafereo J., Wang-Price S., Roddey T., Brizzolara K. (2018). Regional manual therapy and motor control exercise for chronic low back pain: A randomized clinical trial. J. Man. Manip. Ther..

[35] Bello B., Danazumi M.S., Kaka B. (2019). Comparative Effectiveness of 2 Manual Therapy Techniques in the Management of Lumbar Radiculopathy: A Randomized Clinical Trial. J. Chiropr. Med..

[36] Danazumi M.S., Bello B., Yakasai A.M., Kaka B. (2021). Two manual therapy techniques for management of lumbar radiculopathy: A randomized clinical trial. J. Am. Osteopat. Assoc..

[37] Lizis P., Wiater S., Kobza W. (2017). Manual Therapy vs. Kinesiotherapy for People with Lumbar Discopathy: A Pilot Randomized Trial. Rehabil. Sci..

[38] Huber J., Lisiński P., Samborski W., Wytrążek M. (2011). The effect of early isometric exercises on clinical and neurophysiological parameters in patients with sciatica: An interventional randomized single-blinded study. Isokinet. Exerc. Sci..

[39] Kennedy D.J., Noh M.Y. (2011). The Role of Core Stabilization in Lumbosacral Radiculopathy. Phys. Med. Rehabil. Clin. N. Am..

[40] Jeong D.-K., Choi H.-H., Kang J.-I. (2017). Effect of lumbar stabilization exercise on disc herniation index, sacral angle, and functional improvement in patients with lumbar disc herniation. J. Phys. Ther. Sci..

[41] Ye C., Ren J., Zhang J., Wang C., Liu Z., Li F., Sun T. (2015). Comparison of lumbar spine stabilization exercise versus general exercise in young male patients with lumbar disc herniation after 1 year of follow-up. Int. J. Clin. Exp. Med..

[42] Gaowgzeh R.A.M., Chevidikunnan M.F., BinMulayh E.A., Khan F. (2020). Effect of spinal decompression therapy and core stabilization exercises in management of lumbar disc prolapse: A single blind randomized controlled trial. J. Back Musculoskelet. Rehabil..

[43] Kang J.-I., Jeong D.-K., Choi H. (2016). Effect of spinal decompression on the lumbar muscle activity and disk height in patients with herniated intervertebral disk. J. Phys. Ther. Sci..

[44] Demirel A., Yorubulut M., Ergun N. (2017). Regression of lumbar disc herniation by physiotherapy. Does non-surgical spinal decompression therapy make a difference? Double-blind randomized controlled trial. J. Back Musculoskelet. Rehabil..

[45] E Ljunggren A., Weber H., Larsen S. (1984). Autotraction versus manual traction in patients with prolapsed lumbar intervertebral discs. Scand. J. Rehabil. Med..

[46] Chou R., Côté P., Randhawa K., Torres P., Yu H., Nordin M., Hurwitz E.L., Haldeman S., Cedraschi C. (2018). The Global Spine Care Initiative: Applying evidence-based guidelines on the non-invasive management of back and neck pain to low- and middle-income communities. Eur. Spine J..

[47] (2023). WHO Guideline for Non-Surgical Management of Chronic Primary Low Back Pain in Adults in Primary and Community Care Settings. https://www.who.int/publications/i/item/9789240081789.

[48] Cheng Y.-H., Hsu C.-Y., Lin Y.-N. (2020). The effect of mechanical traction on low back pain in patients with herniated intervertebral disks: A systemic review and meta-analysis. Clin. Rehabil..

[49] Vanti C., Panizzolo A., Turone L., A Guccione A., Violante F.S., Pillastrini P., Bertozzi L. (2021). Effectiveness of Mechanical Traction for Lumbar Radiculopathy: A Systematic Review and Meta-Analysis. Phys. Ther..

[50] Wang W., Long F., Wu X., Li S., Lin J. (2022). Clinical Efficacy of Mechanical Traction as Physical Therapy for Lumbar Disc Herniation: A Meta-Analysis. Comput. Math. Methods Med..

[51] Vanti C., Saccardo K., Panizzolo A., Turone L., Guccione A.A., Pillastrini P. (2023). The effects of the addition of mechanical traction to physical therapy on low back pain? A systematic review with meta-analysis. Acta Orthop. Traumatol. Turc..

[52] Amjad F., Mohseni-Bandpei M.A., Gilani S.A., Ahmad A., Hanif A. (2022). Effects of non-surgical decompression therapy in addition to routine physical therapy on pain, range of motion, endurance, functional disability and quality of life versus routine physical therapy alone in patients with lumbar radiculopathy; a randomized controlled trial. BMC Musculoskelet. Disord..

[53] A Harte A., Baxter G.D., Gracey J.H. (2007). The effectiveness of motorised lumbar traction in the management of LBP with lumbo sacral nerve root involvement: A feasibility study. BMC Musculoskelet. Disord..

[54] Kang J., Hyong I. (2017). Changes in Electromyographic Activity of Lumbar Paraspinal Muscles According to Type of Inverted-Spinal-Traction. Wirel. Pers. Commun..

[55] Filiz M.B., Kiliç Z., Uçkun A., Çakir T., Doğan Ş.K., Toraman N.F. (2018). Mechanical traction for lumbar radicular pain: Supine or prone? A randomized controlled trial. Am. J. Phys. Med. Rehabil..

[56] Khan R.R., Riaz S., Rashid S., Sulman M. (2021). Effectiveness of mechanical traction in supine versus prone lying position for lumbosacral radiculopathy. Pak. J. Med. Sci..

[57] Tadano S., Tanabe H., Arai S., Fujino K., Doi T., Akai M. (2019). Lumbar mechanical traction: A biomechanical assessment of change at the lumbar spine. BMC Musculoskelet. Disord..

[58] Chun S.-W., Lim C.-Y., Kim K., Hwang J., Chung S.G. (2017). The relationships between low back pain and lumbar lordosis: A systematic review and meta-analysis. Spine J..

[59] Cardoso L., Khadka N., Dmochowski J.P., Meneses E., Lee K., Kim S., Jin Y., Bikson M. (2022). Computational modeling of posteroanterior lumbar traction by an automated massage bed: Predicting intervertebral disc stresses and deformation. Front. Rehabil. Sci..

[60] Lee C.-H., Heo S.J., Park S.H. (2021). The Real Time Geometric Effect of a Lordotic Curve-Controlled Spinal Traction Device: A Randomized Cross Over Study. Healthcare.

[61] Oakley P.A., Ehsani N.N., Moustafa I.M., Harrison D.E. (2020). Restoring lumbar lordosis: A systematic review of controlled trials utilizing Chiropractic Bio Physics^®^ (CBP^®^) non-surgical approach to increasing lumbar lordosis in the treatment of low back disorders. J. Phys. Ther. Sci..

[62] Moustafa I.M., A Diab A. (2013). Extension traction treatment for patients with discogenic lumbosacral radiculopathy: A randomized controlled trial. Clin. Rehabil..

[63] Lee C.-H., Heo S.J., Park S.H., Jeong H.S., Kim S.-Y. (2019). Functional Changes in Patients and Morphological Changes in the Lumbar Intervertebral Disc after Applying Lordotic Curve-Controlled Traction: A Double-Blind Randomized Controlled Study. Medicina.

[64] Paulk G.P., Harrison D.E. (2004). Management of a Chronic Lumbar Disk Herniation with Chiropractic Biophysics Methods after Failed Chiropractic Manipulative Intervention. J. Manip. Physiol. Ther..

[65] Oakley P.A., Harrison D.E. (2017). Lumbar extension traction alleviates symptoms and facilitates healing of disc herniation/sequestration in 6-weeks, following failed treatment from three previous chiropractors: A CBP^®^ case report with an 8 year follow-up. J. Phys. Ther. Sci..

[66] Yoon Y.-S., Lee J.-H., Lee M., Kim K.-E., Jang H.-Y., Lee K.-J., Bajgai J., Kim C.-S., Cho I.-Y. (2021). Mechanical Changes of the Lumbar Intervertebral Space and Lordotic Angle Caused by Posterior-to-Anterior Traction Using a Spinal Thermal Massage Device in Healthy People. Healthcare.

[67] Peacock M., Douglas S., Nair P. (2023). Neural mobilization in low back and radicular pain: A systematic review. J. Man. Manip. Ther..

[68] Plaza-Manzano G., Cancela-Cilleruelo I., Fernández-de-Las-Peñas C., Cleland J.A., Arias-Buría J.L., Thoomes-de-Graaf M., Ortega-Santiago R. (2020). Effects of adding a neurodynamic mobilization to motor control training in patients with lumbar radiculopathy due to disc herniation: A randomized clinical trial. Am. J. Phys. Med. Rehabil..

[69] Ahmed I., Bandpei M.A.M., Gilani S.A., Ahmad A., Zaidi F. (2022). Effectiveness of Low-Level Laser Therapy in Patients with Discogenic Lumbar Radiculopathy: A Double-Blind Randomized Controlled Trial. J. Healthc. Eng..

[70] Boyraz I., Yildiz A., Koc B., Sarman H. (2015). Comparison of High-Intensity Laser Therapy and Ultrasound Treatment in the Patients with Lumbar Discopathy. BioMed Res. Int..

[71] Unlu Z., Tascı S., Tarhan S., Pabuscu Y., Islak S. (2008). Comparison of 3 Physical Therapy Modalities For Acute Pain in Lumbar Disc Herniation Measured by Clinical Evaluation and Magnetic Resonance Imaging. J. Manip. Physiol. Ther..

[72] Ariel E., Levkovitz Y., Goor-Aryeh I., Motti R. (2022). The effects of TENS, interferential stimulation, and combined interferential stimulation and pulsed ultrasound on patients with disc herniation-induced radicular pain. J. Back Musculoskelet. Rehabil..

[73] Karimi A., Mahmoudzadeh A., Rezaeian Z.S., Dommerholt J. (2016). The effect of dry needling on the radiating pain in subjects with discogenic low-back pain: A randomized control trial. J. Res. Med. Sci..

[74] Dernek B., Adiyeke L., Duymus T.M., Gokcedag A., Kesiktas F.N., Aksoy C. (2018). Efficacy of Trigger Point Injections in Patients with Lumbar Disc Hernia without Indication for Surgery. Asian Spine J..

[75] Hosseini L., Shariat A., Ghaffari M.S., Honarpishe R., Cleland J.A. (2018). The effect of exercise therapy, dry needling, and nonfunctional electrical stimulation on radicular pain: A case report. J. Exerc. Rehabil..

[76] Manchikanti L., Buenaventura R.M., Manchikanti K.N., Ruan X., Gupta S., Smith H.S., Christo P.J., Ward S. (2012). Effectiveness of Therapeutic Lumbar Transforaminal Epidural Steroid Injections in Managing Lumbar Spinal Pain. Pain Physician.

[77] George S.Z., Fritz J.M., Silfies S.P., Schneider M.J., Beneciuk J.M., Lentz T.A., Gilliam J.R., Hendren S., Norman K.S. (2021). Interventions for the management of acute and chronic low back pain: Revision 2021: Clinical practice guidelines linked to the international classification of functioning, disability and health from the academy of orthopaedic physical therapy of the American Physical Therapy Association. J. Orthop. Sports Phys. Ther..

[78] Kreiner D.S., Matz P., Bono C.M., Cho C.H., Easa J.E., Ghiselli G., Ghogawala Z., Reitman C.A., Resnick D.K., Watters W.C. (2020). Guideline summary review: An evidence-based clinical guideline for the diagnosis and treatment of low back pain. Spine J..

[79] Vialle L.R., Vialle E.N., Henao J.E., Giraldo G. (2010). Lumbar disc herniation. Rev. Bras. Ortop. (Engl. Ed.).

[80] Kuligowski T., Skrzek A., Cieślik B. (2021). Manual Therapy in Cervical and Lumbar Radiculopathy: A Systematic Review of the Literature. Int. J. Environ. Res. Public Health.

